# Transferrin receptor targeting segment T7 containing peptide gene delivery vectors for efficient transfection of brain tumor cells

**DOI:** 10.1080/10717544.2022.2102696

**Published:** 2022-07-22

**Authors:** Ziyao Kang, Chunlan Zeng, Long Tian, Taoran Wang, Sen Yang, Qin Cheng, Jing Zhang, Qingbin Meng, Changhao Zhang, Zhao Meng

**Affiliations:** a State Key Laboratory of Toxicology and Medical Countermeasures, Beijing Institute of Pharmacology and Toxicology, Beijing, China; b Key Laboratory of Structure-Based Drug Design and Discovery of the Ministry of Education, Shenyang Pharmaceutical University, Shenyang, China; c Anhui Institute for Food and Drug Control, Baohe, Hefei, China; d Key Laboratory of Natural Medicines of the Changbai Mountain, Ministry of Education, Yanbian University College of Pharmacy, Yanji, Jilin, China

**Keywords:** Peptide vectors, gene delivery, blood brain barrier

## Abstract

Successful gene therapy for brain tumors are often limited by two important factors, the existence of blood brain barrier (BBB) and inefficient transfection of brain tumor cells. In this study, we designed a series of peptide-based gene delivery vectors decorated with T7 segment for binding the transferrin (Tf) receptors which were highly expressed on brain tumor cells, and evaluated their ability of gene delivery. The physicochemical properties of peptide vectors or peptide/DNA complexes were studied as well. The *in vitro* transfection efficiency was investigated in normal and glioma cell lines. Among these complexes, PT-02/DNA complexes showed the highest transfection efficiency in glioma cells and low cytotoxicity in normal cell lines, and it could transport DNA across the BBB model *in vitro*. Furthermore, PT-02/DNA could deliver pIRES2-EGFP into the brain site of zebrafish *in vivo*. The designed peptide vectors offered a promising way for glioma gene therapy.

## 1. Introduction

Glioma is considered to be one of the most malignant tumors with poor prognosis (Wang et al., 2022). Most patients survived less than 15 months after being diagnosed. And a 5 year survival rate of the patients is only 4–5% (Khaddour et al., 2020). Chemotherapy is the most common choice for the patient after surgical resection of glioma. However, the clinical curative effect of drug treatment remains unsatisfying. Because of the special structure of brain and glioma cells, chemotherapeutic drugs could not reach the target site to eradicate or suppress the growth of the glioma cells. Thus, it is urgent to develop desirable methods or delivery system to treat the glioma.

Over the past three decades, gene therapy has been considered as an attractive method to treat many refractory diseases, such as cancer, monogenetic diseases, cardiovascular diseases, and AIDS (Junge et al., 2015; Sun et al., 2019; Deng et al., 2020; Cornu et al., 2021). However, the cellular uptake of free oligonucleotides and plasmid DNA is hindered by their instability under physiological conditions and inability to penetrate different kinds of membranes (Qi et al., 2012; Chen et al., 2015). The ‘Achilles’ Heel’ for the successful gene delivery is the lack of efficient vectors to deliver therapeutic gene to the targeting sites and simultaneously protect them from clearing by circulating system rapidly. In the past several years, the development of nanoparticle (NP) as delivery vector has provided a promising way to solve this problem (Ruan et al., 2021). Various kinds of nanoparticle (NP)-based drug delivery systems have been developed to deliver cargos to target site (Gao et al., 2016). As one of the nano vectors, peptide-based vectors with multiple functional segments can be rationally designed and synthesized easily, ultimately aiming to enhance their transfection efficiency (Meng et al., 2017; Yang et al., 2018).

Cell penetration peptides (CPPs) are short peptides with the ability to penetrate biological membranes and delivery various cargos into cells. The TAT peptide (GRKKRRQRRR), which takes effect in the process of cellular uptake (Green et al., 1989), could condense DNA by electrostatic interactions and deliver exogenous DNA across the plasma membranes effectively (Torchilin, 2008). Although the CPPs could deliver DNA into even any kind of cells, there are still some obstacles restricting the delivery efficiency. It is generally recognized that a large part of vectors with cargos will be degraded in endosomes or lysosomes after the complexes uptake by cells. Therefore, it is necessary to improve the ability of the vectors to escape from endosome. Interacting with endosomal membrane or offering a ‘‘proton sponge’’ to destroy the endosomal membrane could help the cargos escape from the endosome (Lo & Wang, 2008). The imidazole group of histidine which plays a role as ‘proton sponge’ can absorb protons in acidic environment, leading to the swelling and rupture of the endosome. In our previous work, histidine-enriched peptides including C_18_-C(LLHH)_3_C-TAT and TAT-H_6_-K(C_18_)-YIGSR could achieve effective gene transfection, the transfection efficiency of which was 3-fold and 7-fold higher than that of Lipofectamine® (Lipo 2000) in 293 T cells, respectively (Meng et al., 2017; 2018). Otherwise, lack of cell selectivity may reduce the amount of the CPPs as well as the cargos entering the target cells. Improving the selectivity of CPPs facilitates the delivery of cargos to target cells. Thus, it is reasonable that combined with targeting segment and endosomal escape segment may increase the efficiency of CPPs.

To achieve brain tumor gene delivery, the gene molecules must be delivered across the blood-brain barrier (BBB) and transfect into brain tumor cells. On the one hand, BBB as a physical barrier is believed to be one of the most compact structures in the body (Yue et al., 2014). Brain capillary endothelial cells are the major components of this barrier and they form a guard that keeps extraneous substances out. Therefore, it is difficult to deliver therapeutic agents into the brain through noninvasive systemic administration (Pahuja et al., 2015). On the other hand, neuronal cells are difficult to be transfected primarily because of their special polarized and elongated morphology. The post-mitotic stage of neuronal cells also makes it difficult to translocate DNA into the nucleus (Zabner et al., 1995; Pérez-Martínez et al., 2011). Transferrin receptors (TfRs) have been reported to be overexpressed on brain capillaries endothelial cells and many malignant tumor cells, such as brain tumor cells (Zhang et al., 2012). The application of transferrin (Tf) as a targeting motif has been hampered owing to the competitive inhibition of endogenous Tf (Ulbrich et al., 2009). The peptide T7 (HAIYPRH), identified in a phage display screen, is an ideal exogenous targeting ligand for TfRs since its binding site on TfR is entirely different from Tf (Zhang et al., 2018; He et al., 2022). T7 was easy to synthesize and not competed by endogenous transferrin with small steric hindrance and great stability (Oh et al., 2009). Kuang et al. designed a gene delivery vectors DGL-PEG-T7 containing dendrigraft poly-L-lysines (DGLs), poly(ethylene glycol) (PEG) and T7 segments. The DGL-PEG-T7/psiLuc could achieve 2.17-fold silencing ability *in vivo* than vectors without T7 segments (Kuang et al., 2013). Cai et al. designed a dendrigraft poly-L-lysines (DGL)-based delivery system which could programmatically target BBB and neurons. The system contained DGL skeleton, D peptide that could ameliorate the neuropathology, and Tet1 segment which could bind to the highly expressed sphingomyelin and ganglioside GT1B on neurons. Transferrin receptor targeting segment T7 was also conjugated with the delivery system by an acid response as well as a long bifunctional polyethylene glycol (PEG) linker. The system programmatically targeted BBB and neurons, and significantly enhanced the intracephalic drug accumulation and AD treatment efficacy (Cai et al., 2020). However, T7 segment can’t combine with the therapeutic reagent and achieve endosomal escape. The efficient brain tumor gene delivery can be achieved by rational design and arrangement of the functional segments.

In this study, several peptide vectors containing different functional segments were designed. Among them, T7 was selected as a dual-targeting sequence that could deliver vector/DNA complexes across BBB and selectively bind to the TfRs overexpressed on brain tumor cells. TAT (GRKKRRQRRR) was selected as the cell penetrating segment. Two histidine-enriched segments ((LLHH)_3_ and H_6_) were chosen to help the endosomal escape. A stearyl group (C_18_) was conjugated to the side chain of a lysine, which could enhance the cellular uptake and endosomal escape of vector/DNA complexes by facilitating their interaction with membranes (Tönges et al., 2006; Toriyabe et al., 2013). The physicochemical properties and the cytotoxicity of peptide/DNA complexes were studied. In addition, their transfection efficiency in glioma cells and transcytosis across the BBB model were also evaluated in detail.

## 2. Materials and methods

### 2.1. Materials

N-(Fluorenyl-9-methoxycarbonyl) (Fmoc)-protected L-amino acids, 2-(1H-Benzotriazol-1-yl)-1, 1, 3, 3-tetramethyluronium hexafluorophosphate (HBTU) and hydroxybenzotriazole (HOBT) were purchased from GL Biochem Ltd (Shanghai, China). Rink amide resin was purchased from Nankai Hecheng (Tianjin, China). N, N-Diisopropylethylamine (DIEA), trifluoroacetic acid (TFA), thioanisole, ethanedithiol, anisole, hydrazine hydrate, N-Methyl pyrrolidone (NMP), N, N-Dimethyl formaide (DMF) were purchased from J & K Scientific (Beijing, China). F12K medium and MEM medium were purchased from MACGENE (Beijing, China). Dulbecco’s modified Eagle medium (DMEM), Fetal bovine serum (FBS), Horse Serum (HS), Penicillin-streptomycin, Phosphate-buffered saline (PBS), and 0.25% Trypsin-EDTA were purchased from Gibco (Thermo Fisher Scientific). YOYO-1 (491/501) intercalating dye and Lipofectamine 2000 (Lipo 2000) were purchased from Invitrogen (CA, USA). A pGL3 control vector, a Dual-Glo Luciferase Assay System, and a CellTiter96® Aqueous One Solution cell proliferation assay were purchased from Promega (WI, USA). A pIRES2-EGFP plasmid was obtained from Sino Biological Inc. A Bradford protein assay kit was purchased from Beijing Solarbio Science & Technology Co. Ltd (Beijing, China). A Cell Counting Kit-8 (CCK-8) was purchased from Dojindo Molecular Technologies, Inc. DNase I was obtained from BBI Life Sciences Corporation. DAPI was purchased from Roche (Basel, Switzerland). Lyso-Tracker Red was purchased from Beyotime (Shanghai, China). Hochest, CPZ was purchased from TCI (Tokyo, Japan). MβCD was purchased from Adamas (Shanghai, China). Amiloride was purchased from Sigma-Aldrich (Shanghai, China).

### 2.2. Peptide synthesis and purification

All peptides were synthesized using a Liberty automated microwave peptide synthesizer (CEM Co., Matthews, NC) together with a standard solid-phase Fmoc chemistry protocol. Rink amide resin with a loading capacity of 0.44 mmol/g was used as a solid phase to obtain C-terminally amidated peptides. Coupling of the amino acids was achieved using HBTU in DMF as an activator and DIEA in NMP as an active base. A 20% (v/v) solution of piperidine in DMF was added to the resin for deprotection. After every coupling or deprotection, the resin was washed with DMF and DCM for three times respect. Amino acids were individually coupled to Rink amide resin, followed by the amino group in the side chain of lysine conjugation of stearic acid. Final cleavage was performed with 10 mL of TFA (90%)/thioanisole (5%)/ethanedithiol (3%)/anisole (2%) for 3 h at room temperature. The products were purified by preparative reverse-phase high-performance liquid chromatography (RP-HPLC) using a C8 column (Waters, USA). All peptides were purified to >95% purity. The molecular weights (MWs) of the peptides were determined by Electrospray Ionization Quadrupole-Time of Flight mass spectrometry (MALDI-TOF-MS; Waters Micromass Q-TOF Micro Mass Spectrometer).

### 2.3. CD measurements

Peptide was dissolved in a 50% (v/v) solution of trifluoroethanol/PBS with the final concentrations of 50 μmol L ^− 1^ to imitate the cell membrane environment. CD experiments were performed with a Bio-Logic MOS-450 (Claix, France) at room temperature. The experimental conditions were set up as follows: wavelength, 190–260 nm; speed of 50 nm min^−1^, time response of 2 s, resolution of 0.5 nm, bandwidth of 4.0 nm, and cell path length of 1.0 mm. All spectra were converted to a uniform scale after subtraction of the background. The recorded curves were smoothed with standard parameters.

### 2.4. Preparation of vector/DNA complexes

DNA (pGL3, 1.0 μg) was diluted in 50 μL of PBS and the corresponding amount of peptide was dissolved in 50 μL of PBS. Peptide solution was added to pGL3 solution drop by drop slowly. Similarly, as a positive control, 1.0 μg of pGL3 (in 100 μL PBS) was mixed with 2.5 μL of Lipo 2000 (1.0 mg mL ^− 1^). The mixtures were vortexed for 30 s, and then they were incubated at 37 °C for 30 min to form complexes. Peptides/pGL3 complexes with various charge ratios (N/P) were freshly prepared and diluted to appropriate concentrations in PBS (pH 7.4) or corresponding medium.

### 2.5. Agarose gel electrophoresis assay

Peptide/pGL3 complexes were prepared at N/P ratios ranging from 0 to 4 using 0.5 μg of pGL3 DNA in the peptide solutions. Then the complexes were diluted to 10 μL with PBS. After that, the solution was incubated at 37 °C for 30 min. The solutions were mixed with 2 μL tris-acetate-ethylenediaminetetraacetic acid buffer and then loaded on a 1% (w/v) agarose gel. Electrophoresis was performed at 100 V for 45 min. The gels were stained with 300 mL of ethidium bromide solution for 30 min. Stained pGL3 was imaged under an ultraviolet lamp using a ChampGel 6000 system (Beijing Sage Creation Science and Technology Co., Ltd, Beijing, China).

### 2.6. Dynamic light scattering (DLS) and zeta (ζ) potential measurements

The particle sizes and zeta potentials of peptides/pGL3 complexes were measured by DLS at 25 °C using a Zetasizer Nano ZS90 analyzer (Malvern, UK) with a fixed scattering angle of 90°. Peptides/pGL3 complexes were prepared at N/P ratios ranging from 2 to 6 with 100 μL of a DNA solution (containing 2.0 μg pGL3) and the corresponding amount of a peptide solution. After incubation at 37 °C for 30 min, the mixture was used to measure the size of the peptide/DNA complexes. Then, each of the sample solutions was diluted with deionized water to 1 mL aiming to measure zeta potentials. The measurements of DLS and zeta potentials were both repeated for three times, respectively.

### 2.7. Transmission electron microscopy (TEM)

The morphology of peptides/pGL3 complexes with an N/P ratio of 4 were observed by TEM (Hitachi H-7650, Tokyo, Japan). Complexes were prepared as described above by the addition of 1.0 μg of pGL3 to the appropriate peptide solution. After incubation for 30 min, complexes were diluted to 500 μL with deionized water. Then the peptides/pGL3 complexes were dropped on the carbon coated mesh grid (300-mesh). After 20 min, excess solution on the surface of carbon coated mesh grid was removed by filter paper and then dried in air before measurement.

### 2.8. Cell culture

Human embryonic kidney cells (293 T), mouse fibroblast cell line (L929), human glioblastoma cells (U87), and rat glioma cells (C6) were purchased from Cell Resource Center. The 293 T and L929 cells were grown at 37 °C under 5% CO_2_ in DMEM with 10% FBS and 1% penicillin–streptomycin (10 000 U mL ^− 1^). The U87 cells were grown at 37 °C under 5% CO_2_ in MEM with 10% FBS and 1% penicillin–streptomycin (10 000 U mL ^− 1^). The C6 cells were grown at 37 °C under 5% CO_2_ in F12K with 2.5% FBS, 12.5% HS and 1% penicillin–streptomycin (10 000 U mL ^− 1^).

### 2.9. Cytotoxicity assay

The cytotoxicity of the peptides/pGL3 complexes was assessed in U87 and C6 cells with a CCK-8 kit (Dojindo). After seeded at a density of 1 × 10^4^ cells/well on a 96-well plate, cells were cultured for 24 h in 100 μL medium containing 10% FBS. Peptides/pGL3 complexes were prepared with N/P ratios of 2 to 8 by the addition of 0.2 μg of DNA to the corresponding peptide solution. The mixture in each well was diluted to 100 μL with FBS-free medium. After 24 h incubation, the medium was removed. Then, the peptides/pGL3 complexes with various N/P ratios were added to each well. The medium was removed after 4 h incubation with the cells, and 100 μL fresh medium containing 10% FBS was added to each well. After incubation for 20 h at 37 °C in a 5% CO_2_ atmosphere, 10 μL of CCK-8 reagent was added to each well. The cells were incubation at 37 °C for 2 h, and then the absorbance values were measured at 450 nm using a SpectraMax® M5 microplate reader (Molecular Devices, WI, USA). Cells without complexes were used as a negative control. Lipo 2000/DNA complexes were used as a positive control. The experiment was repeated five times for each sample.

### 2.10. Fluorescence-activated cell sorter (FACS) analysis

U87 and C6 cells were seeded in 6-well plates at a density of 2 × 10^5^ cells per well and were cultured for 24 h. The pGL3 control plasmid (1.0 mg) was incubated with 2.5 mL of YOYO-1 (10 mM) for 30 min at 37 °C to label the DNA. At the same time, to study the mechanism responsible for the cellular uptake of peptide/pGL3 complexes, U87 cells in three wells were pretreated with an endocytosis-specific inhibitor [CPZ (10 mg/mL), amiloride (50 mM), or MbCD, (5 mM)] for 30 min. YOYO-1 labeled Peptide/pGL3 complexes were prepared with N/P ratios of 4 by the addition of 1 μg of DNA to the corresponding peptide solution. The cells were treated with YOYO-1 labeled peptides/pGL3 complexes and incubated for 4 h. Every well was washed with PBS for three times. 0.25% (w/v) trypsin and 0.02% (w/v) EDTA solution were added 200 μL per well until about 80% of cells were detached from the plates. Then, 800 μL medium containing 10% FBS was added to each well. The samples were collected into 1.5 mL centrifuge tubes and centrifuged for 5 min. Supernatant was removed and the precipitate cells were collected and diluted to 250 μL with PBS. A minimum of 10000 events per sample were analyzed by FACS (Becton Dickinson, NJ, USA) at an excitation/emission ratio of 488 nm/530 nm.

### 2.11. Intracellular localization experiment

Cells (U87 and C6) were seeded into 15 mm glass-bottom culture dishes (NEST) with about 3 × 10^4^ cells per well and incubated for 24 h. The cells were treated with YOYO-1 labeled peptides/pGL3 complexes and incubated for 4 h. Every well was washed with PBS for three times. The dishes fixed with 4% formaldehyde/PBS solution for 10 min, and then the dishes were washed three times again with PBS. After that, the cells were incubated with Triton-X for 5 min and washed with PBS for three times. Thereafter, the cell nucleus was stained with DAPI (2 μg/mL) for 15 min. Cells were washed with PBS for three times. The fluorescence was analyzed by Confocal laser scanning microscopy (CLSM, Nikon, Japan) equipped with a 488 nm argon laser for YOYO-1, a 405 nm diode for DAPI.

For live-cell imaging experiments, the cells were seeded into NEST dishes at a density of 5000 cells per well and were cultured for 24 h. Then, pGL3 control plasmid (1.0 mg) was incubated with 2.5 mL of YOYO-1 (10 mM) for 30 min at 37 °C. The cell dishes were incubated with LysoTracker Red (50 nM) for 50 min and washed for three times. Then, the cell dishes were incubated with Hoechst 33258 (1 μg mL ^− 1^) for 20 min to stain the nuclei and were washed with PBS for three times. Every dish should be added with 100 μL PBS to maintain cell vitality. Thereafter, P-03/pGL3 or Lipo 2000/pGL3 (YOYO-1 labeled) complexes were added to the dishes, and the cells were examined with an UltraVIEW VoX live-cell imaging system (PerkinElmer, USA) equipped with a modular laser system using solid-state laser technology for about 4 h.

### 2.12. Transport across *in vitro* BBB model

Brain microvascular endothelial cells (BMVECs) were isolated from the brain of Sprague Dawley rat (SD rat) (two weeks). A Transwell apparatus (Corning, NY, USA) with a polyester (PET) Transwell-Clear inserts (pore size 0.4 μm; membrane diameter 24 mm) as well as a 6-well plate was used to establish an in vitro BBB model. Each well was seeded with 2 × 10^5^ BMVECs. BMVECs were incubated with ECM medium and the medium should be replaced every two days. After cultured for10 days, the transendothelial electrical resistance (TEER) was monitored using epithelial voltohmmeter to evaluate the cell monolayer integrity. Permeability experiments could be performed when TEER values were >200 Ω· cm^2^. Then C6 cells were seeded in 6-well plates at a density of 2 × 10^5^ cells per well and were cultured for 24 h. Before the experiments, inserts were replaced onto the 6-well plates that were seeded with C6 cells.

To label the DNA, pGL3 control plasmid (1.0 mg) was incubated with 2.5 μL of YOYO-1 (10 mM) for 30 min at 37 °C. Peptide/pGL3 complexes were prepared at N/P ratios of 4 by the addition of 1, 5, 10, 20, 40 μg of DNA to the corresponding peptide solution, respectively. Lipo 2000 was used as a positive control and naked DNA without delivery vectors was used as negative control. The medium in upper and deeper of insert were both removed. Samples were added onto the upper side of insert and F12-K medium without FBS were added onto the deeper side of insert. The BBB models were incubated with samples for 24 h at 37 °C. Then inserts were removed and C6 cells were incubated with fresh medium containing 15% HS and 5% FBS for 24 h. Every well was washed with PBS for three times. The dishes fixed with 4% formaldehyde/PBS solution for 10 min, and were washed for three times with PBS. Then, the cells were incubated with 0.1% Triton X-100 for 5 min and washed with PBS for three times again. Thereafter, the cell nucleus was stained with DAPI (2 μg/mL) for 15 min. Cells were washed with PBS three times. The fluorescence was analyzed by CLSM (C1-Si, Nikon, Japan) equipped with a 488 nm argon laser for YOYO-1, a 405 nm diode laser for DAPI.

### 2.13. *In vitro* transfection efficiency

*In vitro* gene transfection of pGL3 plasmids mediated by peptide-based gene delivery vectors against 293 T, L929, U87 and C6 cells was investigated, and Lipo 2000 was used as a positive control. Cells were seeded onto 24-well plates at 3 × 10^4^ cells/well with 300 μL medium and incubated for 24 h at 37 °C. Peptides/pGL3 complexes were prepared with N/P ratios ranging from 2 to 6 by the addition of 0.3 μg DNA to the corresponding peptide solution. The cells were treated with peptides/pGL3 complexes and incubated for 4 h. Then, the medium was replaced with fresh medium containing 10% FBS. After 44 h, the medium was removed and cells were washed with PBS for three times. Subsequently, cells were incubated with 55 μL 1× Cell Lysates for per well. After 15 min, 100 μL reporter lysis buffer (Pierce) was added to 20 μL supernatant. The luciferase expression was measured by the luciferase assay system according to the manufacturer’s protocol. Luciferase activity was expressed as RLU/mg protein, while relative light units (RLU) were determined by chemiluminometer (Lumat LB9507, EG&G Berthold, Germany) and the total protein concentration was measured through BCA protein assay kit (Pierce).

### 2.14. *In vivo* transfection

Before the eggs were collected, the male and female zebrafish were cultured in a water tank with a divider between them. After removing the divider, the fishes started to mate under the stimulation of light. After fertilization, the eggs were collected, cleaned and preserved in embryo medium (17 mM NaCl, 2 mM KCl, 1.8 mM Ca(NO_3_)_2_, 0.12 mM MgSO_4_, 1.5 mM HEPES buffer pH 7.1–7.3 and 0.6 μM methylene blue) in an incubator under constant light and temperature conditions for 72 h. It was reported that after 72 hours growth, the BBB of zebrafish eggs were mature (Jeong et al., 2008). Embryonic zebrafish were put into a 7 cm culture dish and anesthetized by the addition of tricaine methanesulfonate to the dish with a final concentration of 0.64 mM. The anesthetized zebrafish were placed in the slanting grooves of a silica gel sheet. Then, 10 μL of a PT-02, PT-05 and Lipo 2000/pIRES2-EGFP complexes solution were injected into the zebrafish eggs with an IM 300 microinjection instrument (Narishige, Japan), respectively. The solution of the P-02 and P-05/pIRES2-EGFP complex were prepared from 4 μL of DNA solution (0.25 μg μL ^− 1^) with 6 μL of P-02 or P-05 solution (2 μg μL ^− 1^), respectively. And the Lipo 2000/pIRES2-EGFP complexes were prepared with 1 μg pIRES2-EGFP and 2.5 μL Lipo 2000 (1 mg/mL) as the positive control. After injected, the eggs were then rinsed with the zebrafish embryo medium and incubated in a Petri dish at 28 °C. After 24 h growth, the zebrafish were observed with an UltraVIEW VoX live-cell imaging system (PerkinElmer, USA) equipped with a modular laser system using solid-state laser technology. During the entire period of the experiment, the zebrafish that received microinjections of P-02, P-05 and Lipo 2000/pIRES2-EGFP complexes exhibited no significant differences in death rate, development and teratogenesis in comparison to controls.

### 2.15. Statistical analysis

Data were recorded as the mean and standard deviation (SD) for triplicate or quintuplicate groups (*n* = 3 or 5). Data differences were statistically determined by one-way analysis of variance (ANOVA). A p-value of ≤0.05 was considered to indicate an insignificant difference. A p-value of ≤0.01 was considered to represent a significant difference. A p-value of ≤0.001 was considered to indicate a highly significant difference.

## 3. Results and discussion

### 3.1. Design, synthesis and characterization of peptides

The multifunctional peptide-based gene delivery vectors consisting of TAT, histidine-rich endosomal disruption segment, stearic acid and T7 segment were designed. TAT, a cationic-rich CPP, can bind with negatively-charged plasmid *via* electrostatic interaction. In addition, it can help cellular uptake through endocytosis. H_6_, a histidine-rich domain, can provide protons buffer ability due to its imidazole groups. The imidazole group of histidine has a pKa of ∼6.0 and thus can absorb protons in the acidic environment of the endosome, leading to osmotic swelling and membrane disruption. Furthermore, (LLHH)_3_ was used as the endosomal escape segment in some of the peptide vectors. In this segment, leucine and histidine located at different flanks of α-helix. Leucine residues, which located at hydrophobic flank, not only helped interact with lipid bilayers, but aided the escape from endosomes. The histidine residues located at the hydrophilic flank enhanced the interaction with DNA and provided protons buffer ability to help the endosomal escape. Stearic acid could provide a hydrophobic group to strengthen the interaction with lipid bilayer, thus contributing to cell membrane penetration and endosomal escape. To improve the cellular uptake, a receptor-binding peptide T7 (HAIYPRH) was incorporated into the vectors to bind the TfR on the brain tumor cells. It was reported that the targeting motif in the peptide-based vectors could improve the ability of cell-targeting (Meng et al., 2018; Ding et al., 2021). TfR is highly expressed on brain capillaries endothelial cells and brain cancer cells. Therefore, transferrin family ligands can transport cross the BBB to achieve brain targeting delivery (Ulbrich et al., 2009; Zhang et al., 2012).

Here, five peptide sequences were designed and synthesized, named PT-01 to PT-05 (the sequences are shown in Table 1), respectively. To compare endosomal escape efficiency, H_6_ was introduced in PT-01, PT-03 and (LLHH)_3_ was introduced in PT-02, PT-04, and PT-05, respectively. In addition, to verify the targeting ability of T7, its sequence order was changed as PIHRYHA in PT-03 and PT-04, while PT-05 was designed without T7 segment. All peptides were purified and analyzed by HPLC (purity ≥ 95%). The MWs of the peptides were confirmed by MALDI-TOF-MS. The data are shown in Figures S4 and S5.

**Table 1.** Sequences and molecular weights of peptides.

Compounds	Peptide sequence	Molecμlar weight	
		Calcμlated	Measured
PT-01	GRKKRRQRRR-HHHHHH-K(C_18_)-HAIYPRH	3530.19	3527.82
PT-02	GRKKRRQRRR-LLHHLLHHLLHH-K(C18)-HAIYPRH	4209.13	4206.18
PT-03	GRKKRRQRRR-HHHHHH-K(C18)-PIHRYHA	3530.19	3528.07
PT-04	GRKKRRQRRR-LLHHLLHHLLHH-K(C18)-PIHRYHA	4209.13	4206.00
PT-05	GRKKRRQRRR-LLHHLLHHLLHH-K(C18)	3334.14	3332.37

**Figure 1.**
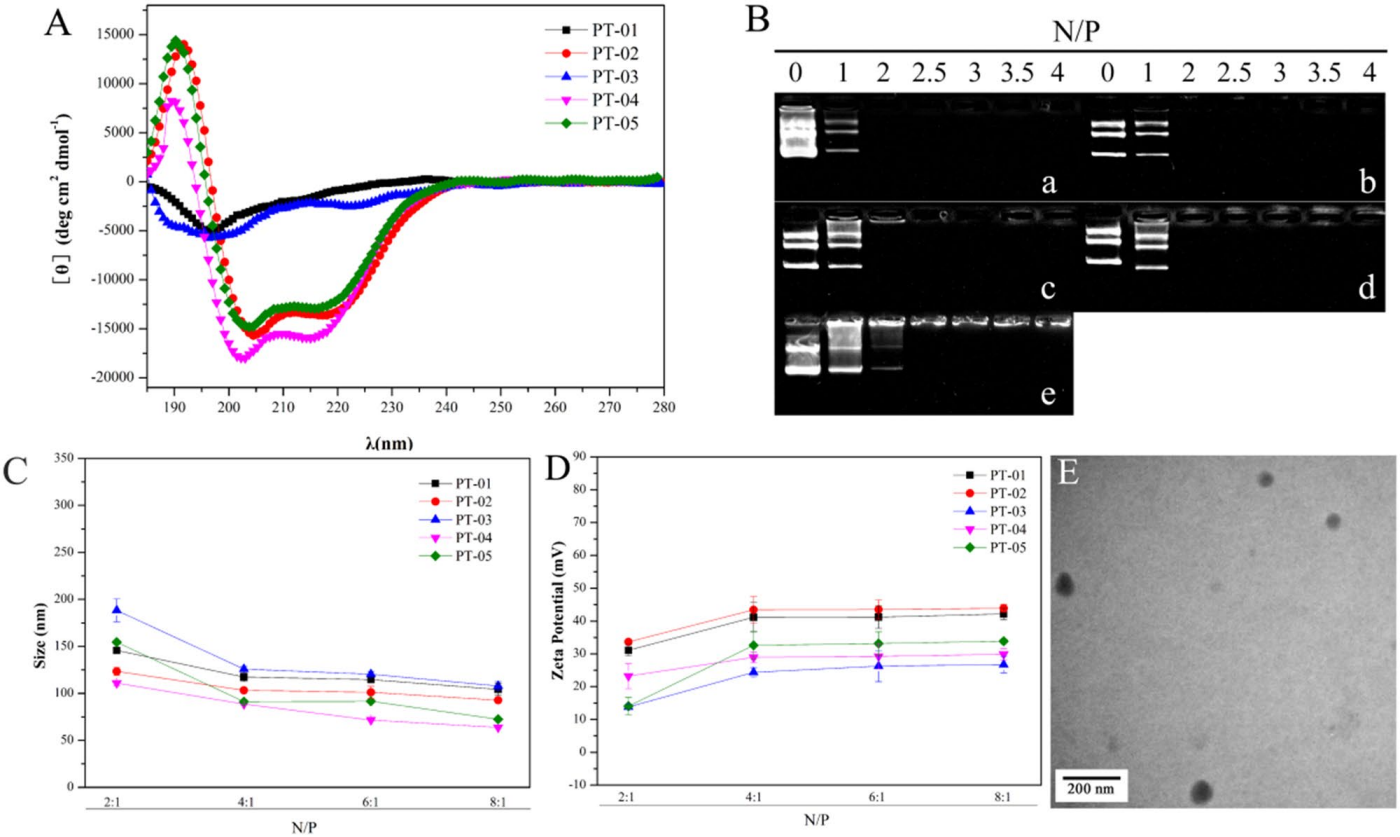
(A) The CD spectra of all peptide vectors at a concentration of 50 μmol L^−1^ in a 50% solution of trifluoroethanol/PBS. (B) Agarose gel electrophoresis assay of (a) PT-01, (b) PT-02, (c) PT-03, (d) PT-04 and (e) PT-05 with different N/P ratios. (C) Particle size and (D) zeta potential of peptide/pGL3 complexes with different N/P ratios. (E) TEM images of PT-02/pGL3 complex with an N/P ratio of 4. The scale bar represents 200 nm.

The α-helical conformation had been proved to facilitate peptides/DNA complexes to interact with lipid bilayers and escape from endosome (Kabelka & Vácha, 2021). Compared with H_6_, (LLHH)_3_ segment with higher α-helical contents were considered to contribute to the interaction with lipid bilayers and escape from endosome. The spectra showed a minimal absorbance at 208 nm and a pronounced shoulder at 220 nm (Figure 1A), which were typical characteristics of α-helical conformation. As shown in Figure 1(A), the α-helicity of PT-01 and PT-03 were lower than 10%. The α-helicity of PT-02 was 39.86% which was much higher than that of PT-01. The results confirmed that the (LLHH)_3_ improved the α-helical conformation of peptides. Furthermore, PT-02 exhibited higher α-helicity than PT-05 with an α-helicity of 36.62%. When T7 was introduced into the peptides, the α-helicity increased slightly, suggesting that T7 segment did not influence the conformation of α-helix. The α-helicity of P-04 was 38.05% which was close to that of P-02 and P-05. The α-helicity of each peptide vectors was shown in Table S1.

The DNA-binding efficiency of peptide-based carriers is the first key point in the delivery process. In this experiment, agarose gel electrophoresis was used to evaluate the appropriate N/P value of the binary complex to ensure that DNA was completely loaded. Peptides/DNA complexes were formed by electrostatic interaction between the cationic peptide and negatively charged DNA. When the DNA was not completely loaded by peptide-based carriers, the dissociated DNA would move to the positive electrode in the agarose gel, and displayed white strip under ultraviolet lamp after being stained by EB. As shown in Figure 1(B), almost all the peptides were able to bind DNA completely at an N/P ratio of 2 except PT-05 which retarded the DNA migration at an N/P ratio of 2.5, indicating that the presence of the targeting fragment could increase the DNA-binding ability of peptide vectors to a certain extent. According to the results, the peptide vectors had the suitable DNA binding ability which was beneficial for the formation of peptides/DNA complexes.

The particle size and zeta potential of peptide/DNA complexes would greatly affect the cellular uptake ability, uptake mechanism, membrane permeability and transfection efficiency. We measured the particle size and zeta potential of peptide/DNA complexes at N/P ratios ranging from 2 to 8 using a laser particle size analyzer. As shown in Figure 1(C), the sizes of peptide/DNA complexes mentioned above decreased sharply when the N/P ratio changed from 2 to 4 and changed slightly when the N/P ratio increased from 4 to 8. The results indicated that all the peptide vectors could form stable nanoparticles at the N/P ratio of 4 because of the slightly change of the particle size when the N/P ratio continued to increase. And the size of all peptide vectors was in the range of 80-130 nm at the N/P ratio of 4.

Proper positive charge on the surface of particles could enhance the stability of complexes by charge repulsion, and also facilitate cellular uptake. The zeta potential of all peptide/DNA complexes increased when the N/P ratio changed from 2 to 4 and tended to be stable when the N/P ratio was over 4 (Figure 1(D)). The above results suggested that when the N/P ratio was larger than 4, the peptide/DNA complexes could form stable self-assembled particles with nanoscale and started to reach stable state. The particle size was in the range of 60 to 120 nm.

The morphology of the peptide/DNA complexes at an optimal N/P ratio of 4 was observed by TEM. As shown in Figure 1(E), the complexes of PT-02/DNA exhibited a uniform spherical shape, and the particle size was mainly distributed at 70–90 nm. The particle size measured by TEM was smaller than that from DLS. The reason was that the size observed by TEM was in the dried state, while the complex swelled in an aqueous solution when measured by DLS.

### 3.2. Cytotoxicity

Biocompatibility is necessary for eligible gene delivery vectors. To evaluate the cytotoxicity of peptides/DNA complexes, a CCK-8 assay was used in C6 and U87 cells, respectively. As shown in Figure S1, the cell viability almost remained above 90% and exhibited significantly lower cytotoxicity than Lipo 2000 which had a cell viability of about 80%. All the peptides didn’t show significant cytotoxicity in both C6 and U87 cells.

### 3.3. Cellular uptake and translocation of peptides/DNA complexes

**Figure 2.**
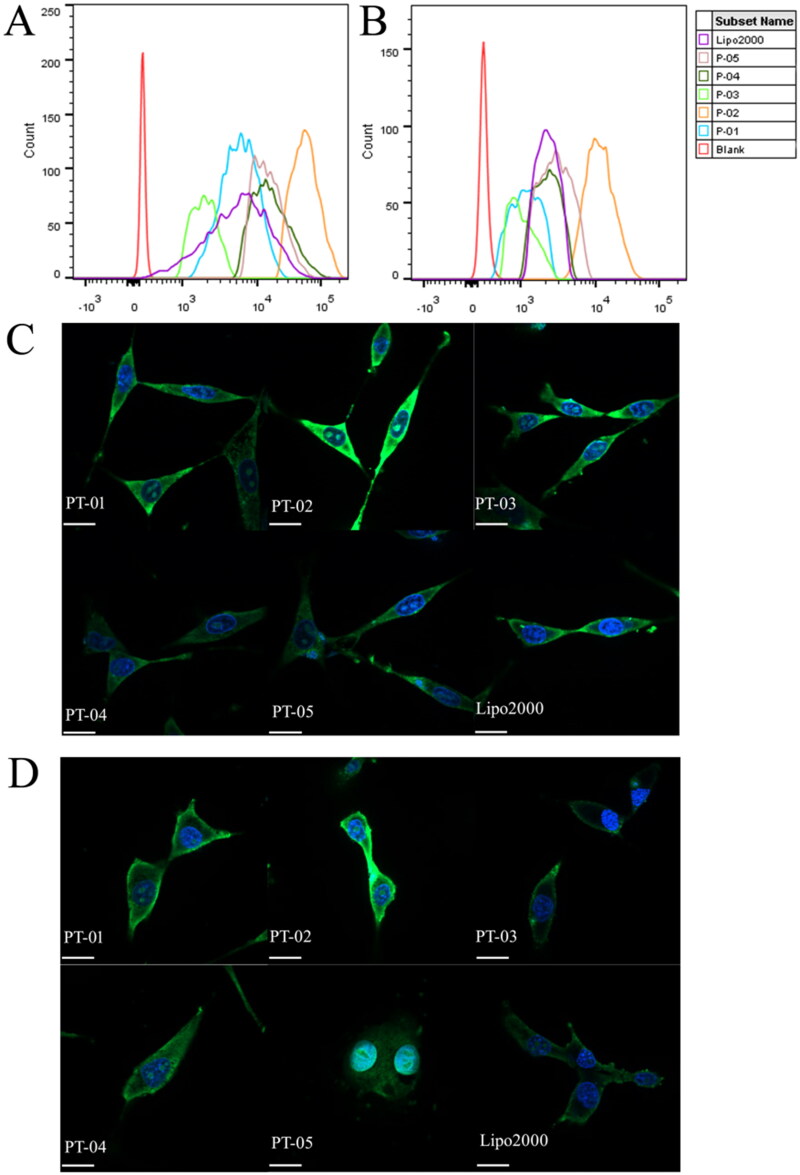
FACS assays of cellular uptake of different peptide/DNA complexes with an N/P ratio of 4 and Lipo 2000/DNA complexes into C6 cells (A) and U87 cells (B). CLSM images of cellular uptake of peptide/DNA complexes with an N/P ratio of 4 into C6 (C) and U87 (D) cells after incubation for 4 h. The pGL3 plasmid was labeled with YOYO-1. The nuclei were stained with DAPI. The scale bar represents 20 μm.

Cellular uptake, endosome escape and nuclear transfer are important processes for gene transfection. FACS and CLSM were used to evaluate the cellular uptake of peptides/pGL3 (YOYO-1 labeled) complexes at the N/P ratio of 4. Figure 2(A) and (B) presented the amount of peptides/pGL3 (YOYO-1 labeled) complexes uptake by C6 and U87 cells, respectively. The cellular uptake mediated by PT-02 was generally higher than other peptide-based vectors and Lipo 2000 in both cell lines. PT-03, PT-04 and PT-05, without T7 segment or with pseudo-T7, exhibited lower cellular uptake efficiency compared with PT-02, suggesting that the effect of T7 on cellular uptake cannot be neglected. The peptide vector PT-01, whose endosomal escape segment was H_6_, also showed poor uptake efficiency. It was proved that (LLHH)_3_ segment could facilitate the transportation of peptides/DNA complexes compared with H_6_.

In order to observe the cellular uptake and the intracellular location of peptides/pGL3 (YOYO-1 labeled) complexes more visually, the process of cellular uptake was observed via CLSM. Cell nucleus stained by DAPI and pGL3 plasmid labeled by YOYO-1 was in blue and green, respectively. As shown in Figure 2(C) and (D), all peptides/pGL3 complexes were internalized into C6 and U87 cells after incubation for 4 h. PT-02/pGL3 (YOYO-1 labeled) complexes showed the strongest fluorescence intensity among these samples and even were localized into the nucleus, which agreed well with the results obtained by FACS.

**Figure 3.**
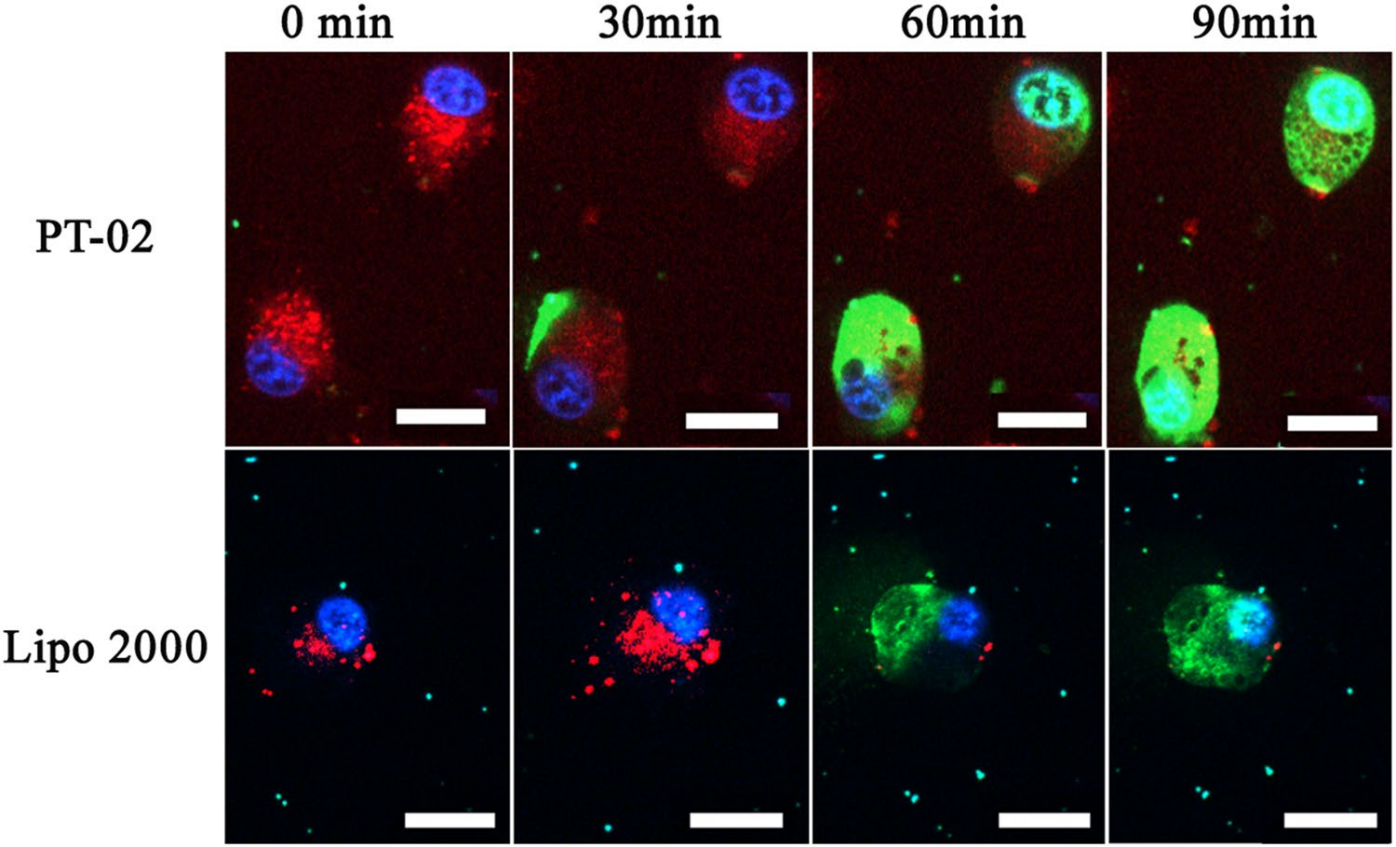
Cellular uptake of P-02 and Lipo 2000/DNA complexes at an N/P ratio of 4 in C6 cells. CLSM images were obtained after incubation with peptide/DNA complexes for about 0, 30, 60 and 90 min. pGL3 plasmid was labeled with YOYO-1. The nuclei were stained with Hoechst 33258. Lysosome/endosomes were stained with Lyso-Tracker Red. The scale bar represents 20 μm.

To observe the whole process of peptide/DNA complexes entry into cells and further study the rate of cellular uptake, live-cell imaging experiments were performed. In these experiments, C6 cells were selected, and PT-02/pGL3 (YOYO-1 labeled) complexes were prepared at an optimal N/P ratio of 4. Lysosome/endosomes stained with Lyso-Tracker Red were shown in red. Nuclei stained with Hoechst 33258 were shown in blue. As shown in Figure 3, after PT-02/pGL3 (YOYO-1 labeled) complexes were incubated with the cells for 30 min, green fluorescence appeared around the lysosome, co-localization with the red lysosome/endosomes showed yellow, indicating the labeled plasmid DNA had been transported into the cytoplasm. At the same time, this phenomenon occurred at about 60 min for Lipo 2000 group. At 60 min, the green color appeared in the nucleus in the PT-02 group. After 90 min, green color completely covered nucleus which were stained with blue, indicating that most of the complexes had entered the nucleus. These results indicated that the PT-02 peptide could deliver pGL3 (YOYO-1 labeled) into cells, escape from the endosomes, and finally enter the nucleus within 90 min. For Lipo 2000, after 90 min only small amount of pGL3 was delivered into the nucleus comparatively.

### 3.4. Cellular uptake mechanisms for the complexes

To clarify the cellular uptake mechanism, three endocytic inhibitors, including CPZ for clathrin-mediated endocytosis, MβCD for caveolin-mediated endocytosis, and amiloride for macropinocytosis, were used to incubate with cells for half an hour before the PT-02/DNA complexes. As shown in Figure S3, after the addition of inhibitors, the uptake of polypeptide/DNA complexes were significantly reduced in U87 and C6 cells indicating that PT-02/DNA complexes could be internalized by cells in various ways.

### 3.5. *In vitro* transfection efficiency

**Figure 4.**
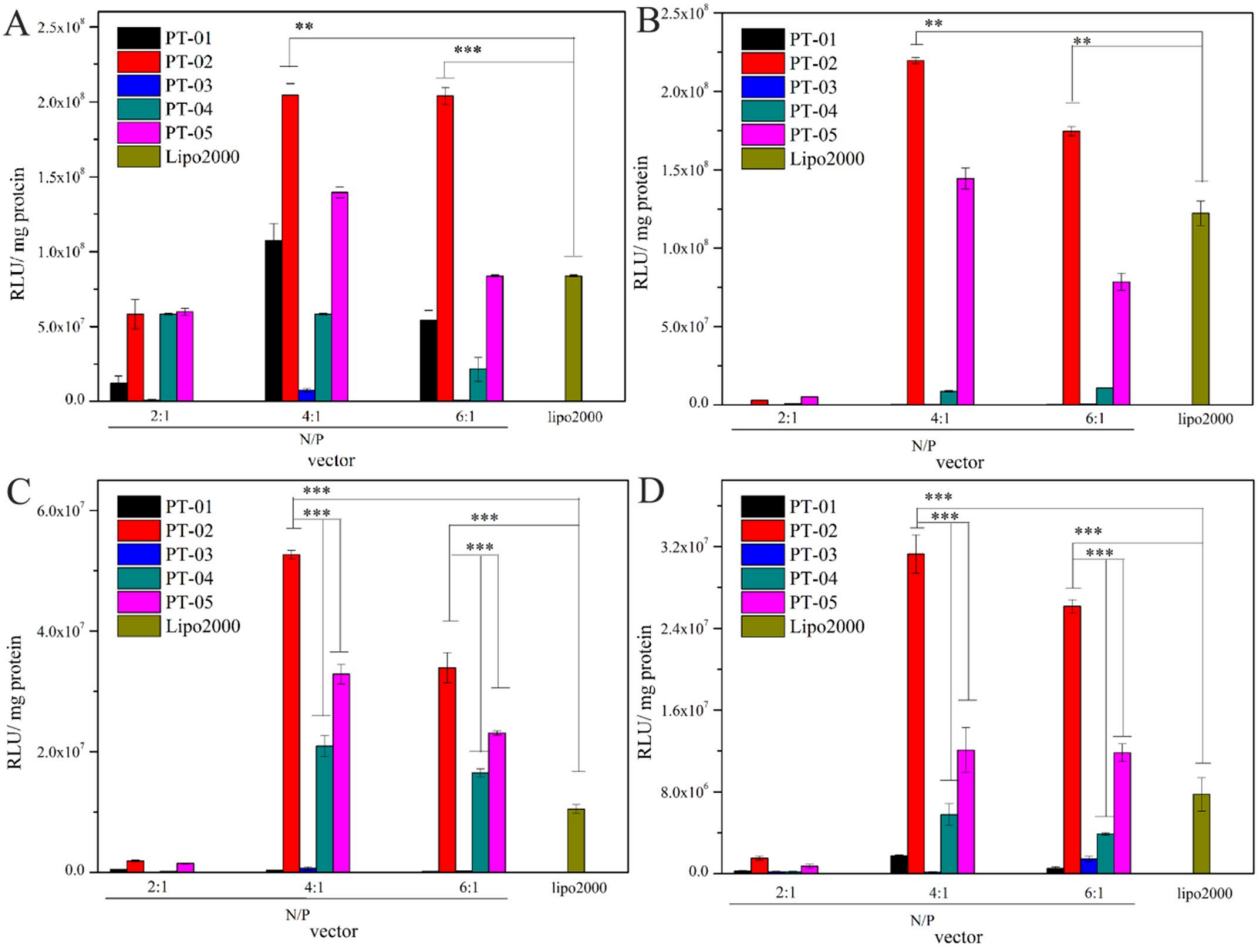
*In vitro* luciferase expression levels in 293 T cells (A), L929 cells (B), C6 cells (C) and U87 cells (D) for peptide/DNA complexes with different N/P ratios. The data are the mean ± SD (*n* = 3). *p < 0.05; **p < 0.01; ***p < 0.005.

The luciferase expression of pGL3 mediated by PT-01 to PT-05 was investigated in normal cells (293 T and L929) and brain tumor cells (C6 and U87), respectively. Herein, Lipo 2000 was used as a positive control. As shown in Figure 4, all the peptides/DNA complexes at the N/P ratio of 2, 4 and 6 could achieve the transfection of pGL3 in both brain tumor cells and normal cells in different degree, among which PT-02 and PT-05 showed greater transfection efficiency than other peptide vectors in almost all cell lines. All the peptides showed the highest transfection efficiency at the N/P ratio of 4 in different cell lines, probably because the peptide and the DNA could form the stable complex under this condition agreeing well with results from DLS and zeta potential. It was reasonable that PT-05 showed the good gene transfection capability, because its sequence was similar to the peptide vector with good transfection efficiency in our previous work (Meng et al., 2017). Compared with the peptides PT-01 and PT-03 containing H_6_ for the endosomal escape segments, P-02 and P-04 (containing (LLHH)_3_) presented higher transfection capability, which could attribute to their relatively high α-helicity. Despite the PT-04 possessed the suitable functional segment and rational arrangement and showed moderate transfection capability, pseudo-T7 segment resulted in the reduced activity comparing with PT-05, let alone PT-02 containing T7 segment in both normal and brain tumor cells. It was worth noting that in C6 and U87 cells, PT-02 showed the 1.6-fold and 2.5-fold transfection efficiency in comparison with PT-05 when the N/P ratio was 4, respectively. However, there was no significant difference between PT-02 and PT-05 in normal cells (293 T and L929). The overexpressed TfR on C6 and U87 cells mediated much higher transfection efficiency of PT-02/pGL3 complexes than 293 T and L929 cells, since T7 could bind the TfR and promote receptor mediated endocytosis leading to the high transfection efficiency. Compared with commercial delivery vector Lipo 2000, the transfection efficiency of PT-02 was 2.1-fold and 1.8-fold higher in 293 T and L929 cells at the N/P ratio of 4, respectively. Notably, it was 4.0-fold and 3.0-fold higher in C6 and U87 cells at N/P ratio of 4, respectively. These results indicated that without T7 or with pseudo-T7 could not possess desirable gene transfection capability in C6 and U87 cells, while PT-02 with T7 segment showed satisfactory transfection efficiency.

Furthermore, C6 and U87 cells were selected to investigate the function of T7 segment in the peptide at the N/P ratio of 4. Therefore, T7 was synthesized and mixed with PT-05 at the molar ratio of 1:1 as a control. As shown in Figure S2, the transfection efficiency of PT-05 mixing with equimolar T7 was slightly higher than that of PT-05, which could attribute to the co-assembly between T7, PT-05 and DNA. In the PT-05/T7 mixture, T7 sequence could also facilitate the cellular uptake of the complexes, to a certain extent. Notably, the transfection efficiency of the mixture was lower than that PT-02. It was supposed that each segment of the PT-02 could play a synergistic role to achieve relatively higher gene transfection efficiency.

### 3.6. Transport across *in vitro* BBB model and *in vivo* transfection

**Figure 5.**
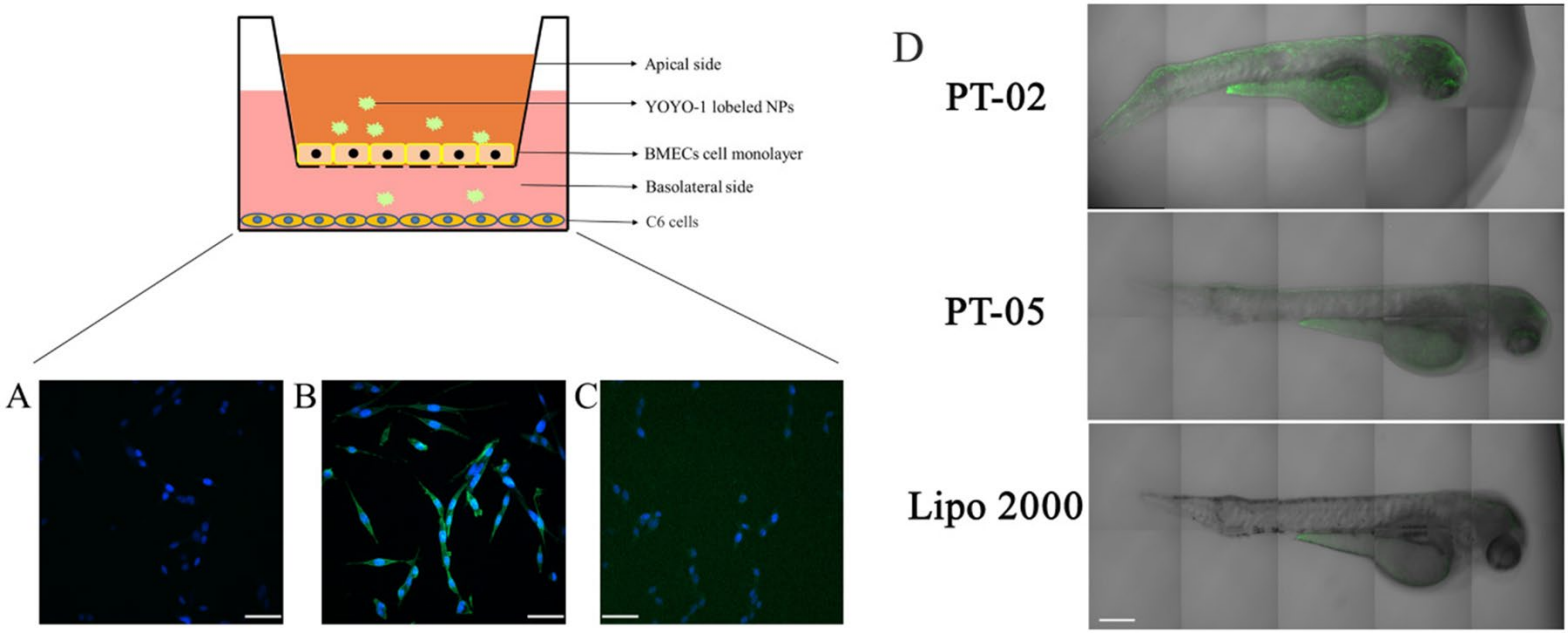
Cellular uptake of 5(A) blank, 5(B) P-02/pGL3 complexes at the N/P ratio of 4, and 5(C) Lipo 2000/pGL3 complexes in C6 cells after crossing the in vitro BBB model presented by CLSM images. The amount of YOYO-1-labeled pGL3 plasmids was 20 μg in each group. DAPI was used to label the nuclei. The scale bar represents 50 μm. And 5(D) CLSM images of zebrafish 24 h after being microinjected with the P-02/pIRES2-EGFP, P-05/pIRES2-EGFP complexes at an N/P ratio of 4 as well as Lipo2000/pIRES2-EGFP complexes. The scale bar represents 200 μm.

The *in vitro* BBB model is capable of mimicking BBB characteristics for a period time and facilitates the effective early-screen of drug candidates designed for targeting delivery of central nervous system. TEER was measured as 200 to 300 Ω·cm^2^ in this study to check the membrane integrity before transport experiments. After the incubation with samples for 24 h, the TEER of BBB model was measured again to make sure that the integrity was not ruptured during transport. No significant difference of TEER was observed, indicating that the cells kept alive during the experiments. The bright-contrast of green fluorescence stands for the amounts of complexes that successfully transport across BBB model and enter in C6 cells. As shown in Figure 5(A), DNA (40 μg) alone could not penetrate the BBB model effectively and enter the cells. Lipo 2000, as a common commercial delivery vector with high transfection efficiency, was difficult to cross the BBB model even at a high concentration (Figure 5(C)). As for PT-02 group, bright green fluorescence could be observed obviously (Figure 5(B)). This result suggested that PT-02/DNA (40 μg) complexes could transport across BBB and achieve cellular uptake.

To investigate whether the peptide vector can achieve gene transfection *in vivo*, zebrafish was used as a model animal due to the similar BBB structure to human beings (Umans & Taylor, 2012; Yang et al., 2015; Li et al., 2017). Additionally, owing to their small size, rapid development, and short life cycle, zebrafish are often used in gene expression studies. More importantly, the zebrafish are transparent and easy to monitor by real-time images (Zon & Peterson, 2005; Hu et al., 2011). As shown in Figure 5(D), the expression of green fluorescent protein was observed in zebrafish brain after being treated with PT-02/pIRES2-EGFP complexes. In contrast, weaker green fluorescence was observed throughout the body and lesser green fluorescence was presented in the brain site of the zebrafish while injected with Lipo 2000/pIRES2-EGFP. For PT-05, the peptide without T7 segment, showed the similar results as Lipo 2000 group. Therefore, the above results proved that P-02 could deliver the EGFP gene across the BBB effectively and achieved gene transfection in the brain of zebrafish successfully.

## 4. Conclusions

In this study, a series of peptide-based gene delivery vectors were designed and synthesized. The results demonstrated that PT-02, which consists of a cationic-rich cell-penetrating peptide (TAT), an endosomal escape sequence (LLHH)_3_, a stearyl group (C_18_) and a targeting sequence (T7), could serve as an effective non-viral vector to transfect brain tumor cells. PT-02 exhibited *α*-helical conformation that could interact with endosomal membrane and improve endosomal escape efficiency of peptides/DNA complexes. At the optimal N/P ratio of 4, PT-02 could bind and condense DNA into stable and uniform sphere-shaped complexes with the size from 70 to 90 nm by self-assembly. PT-02/DNA complexes exhibited a much lower cytotoxicity in comparison to Lip2000/DNA complexes, and cell viability was higher than 90% at the N/P ratio of 4. The cellular uptake of peptide vectors mediated by T7 segment was generally higher than other vectors including vector without T7, pseudo-T7 based vector and Lipo 2000 due to the interaction with TfR over-expressed on brain glioma cells. Compared with Lipo 2000, PT-02 exhibited higher transfection efficiency, which was 2.1-fold higher in 293 T, 1.8-fold higher in L929, 4.0-fold higher in C6 and 3.0-fold higher in U87 cell lines, respectively. Furthermore, PT-02 could effectively deliver DNA across the BBB and achieve brain delivery both *in vitro* and *in vivo*. These findings suggested that PT-02 could serve as a promising peptide-based gene delivery vector for efficient gene delivery in brain tumor cells.

## Supplementary Material

Supplemental Material
