# Supplementary material for: Transferrin receptor targeting segment T7 containing peptide gene delivery vectors for efficient transfection of brain tumor cells
Source: Drug Deliv. 2022 Jul 22;29(1):2375–85. doi: 10.1080/10717544.2022.2102696 (PMC9310815; doi:10.1080/10717544.2022.2102696)
Supplement: Supplemental Material [file IDRD_A_2102696_SM5359.docx]

Supporting Information

Transferrin Receptor Targeting Segment T7 Containing Peptide Gene Delivery Vectors for Efficient Transfection of Brain Tumor Cells

Ziyao Kang^1#^, Chunlan Zeng^1#^, Long Tian^1,2^, Taoran Wang^1^, Sen Yang^1,4^, Qin Cheng^1,3^, Jing Zhang^1,3^, Qingbin Meng^1,3^, Changhao Zhang^*3^ and Zhao Meng^*1^

^1^ State Key Laboratory of Toxicology and Medical Countermeasures, Beijing Institute of Pharmacology and Toxicology, Beijing, 100850, China. E-mail: mengzhao900817@163.com.

^2^ Key Laboratory of Structure-Based Drug Design and Discovery of the Ministry of Education, Shenyang Pharmaceutical University, Shenyang 110016, China.

^3^ Key Laboratory of Natural Medicines of the Changbai Mountain, Ministry of Education, Yanbian University College of Pharmacy, Yanji, Jilin, 133002, China. E-mail: [zhangch@ybu.edu.cn](mailto:zhangch@ybu.edu.cn).

^4^ Anhui Institute for Food and Drug Control, the junction of Baohe Avenue and Urumqi Road, Baohe, Hefei, 230051, China.

* Corresponding authors

# These authors contributed equally to this work.

Tab. S1 The α-helicity of each peptide vectors.

| peptides | α-helicity (%) |
| --- | --- |
| P-01 | 9.87 |
| P-02 | 39.86 |
| P-03 | 8.92 |
| P-04 | 38.05 |
| P-05 | 36.62 |

Fig. S1 Cell viability of C6 cells (A) and U87 cells (B) after treatment with different peptides/DNA complexes at N/P ratios ranging from 2 to 8. The data are the mean ± SD (n = 5).


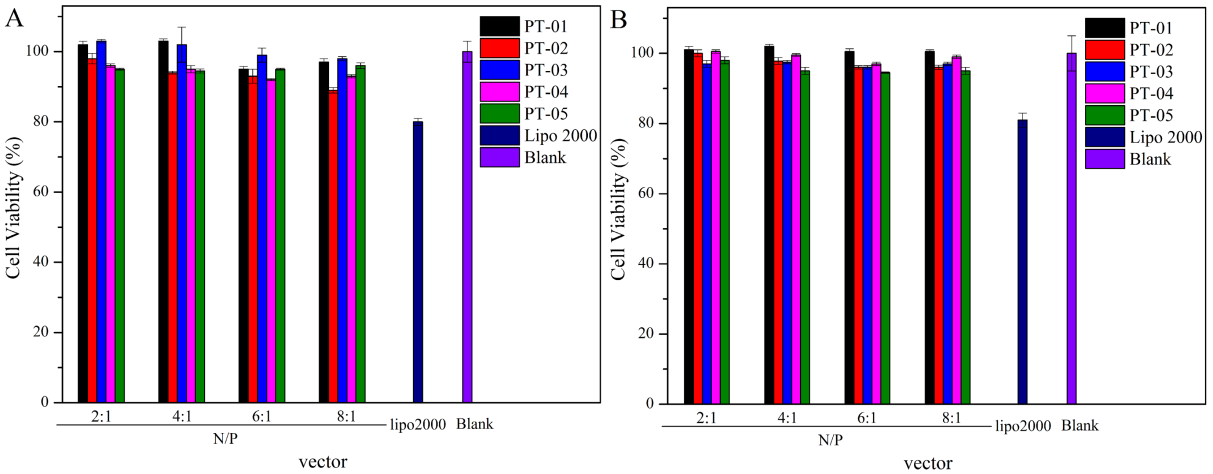


Fig. S2 *In vitro* luciferase expression levels in U87 and C6 cells for peptide/DNA complexes at an N/P ratio of 4.





Fig. S3 FACS assays of cellular uptake pathways of P-02/DNA complexes at an N/P ratio of 4 in U87 (A) and C6 (B) cells with endocytosis-specific inhibitors. And the luciferase expression levels in U87 (A) and C6 (B) cells with endocytosis-specific inhibitors.


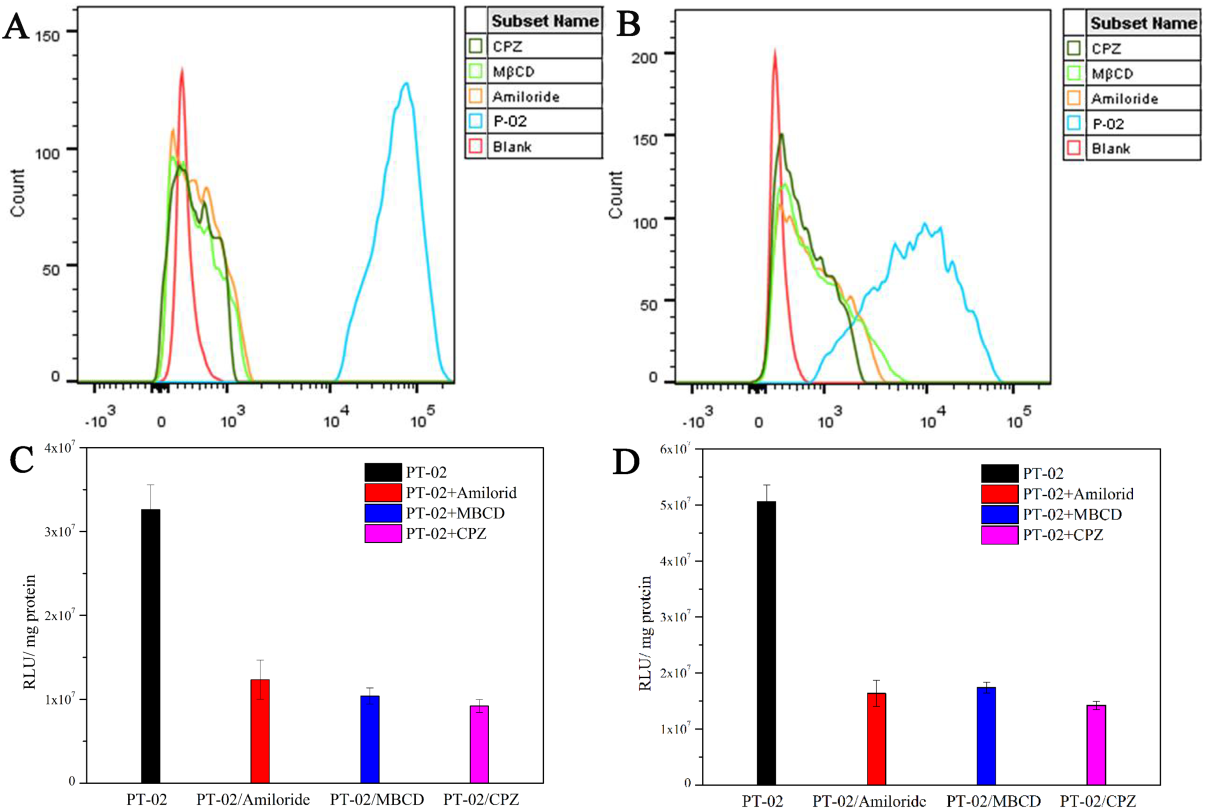


Fig. S4 MALDI-TOF-MS of peptides in this paper.

PT-01: GRKKRRQRRR-HHHHHH-K(C_18_)-HAIYPRH


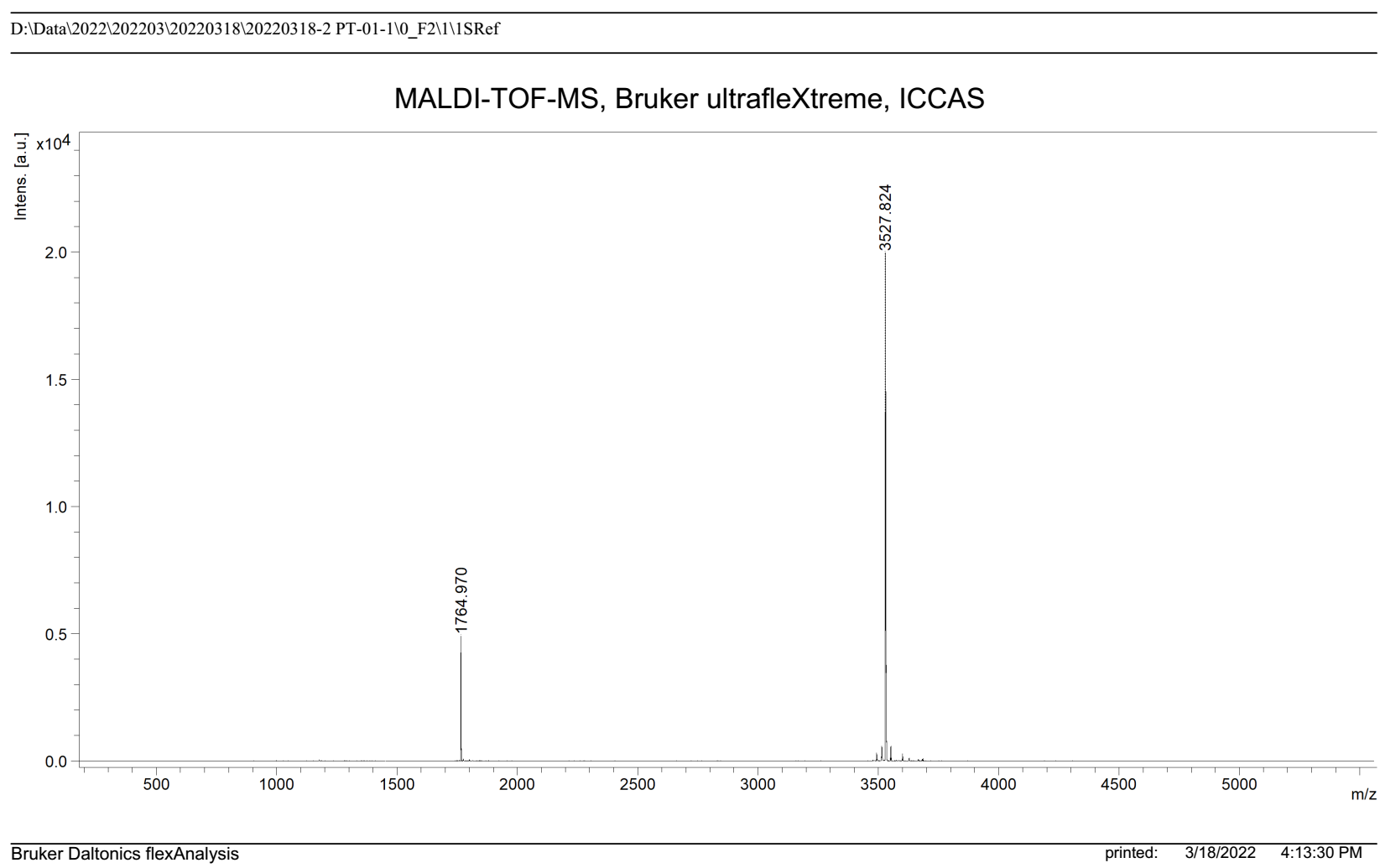


P-02: GRKKRRQRRR-LLHHLLHHLLHH-K(C_18_)-HAIYPRH


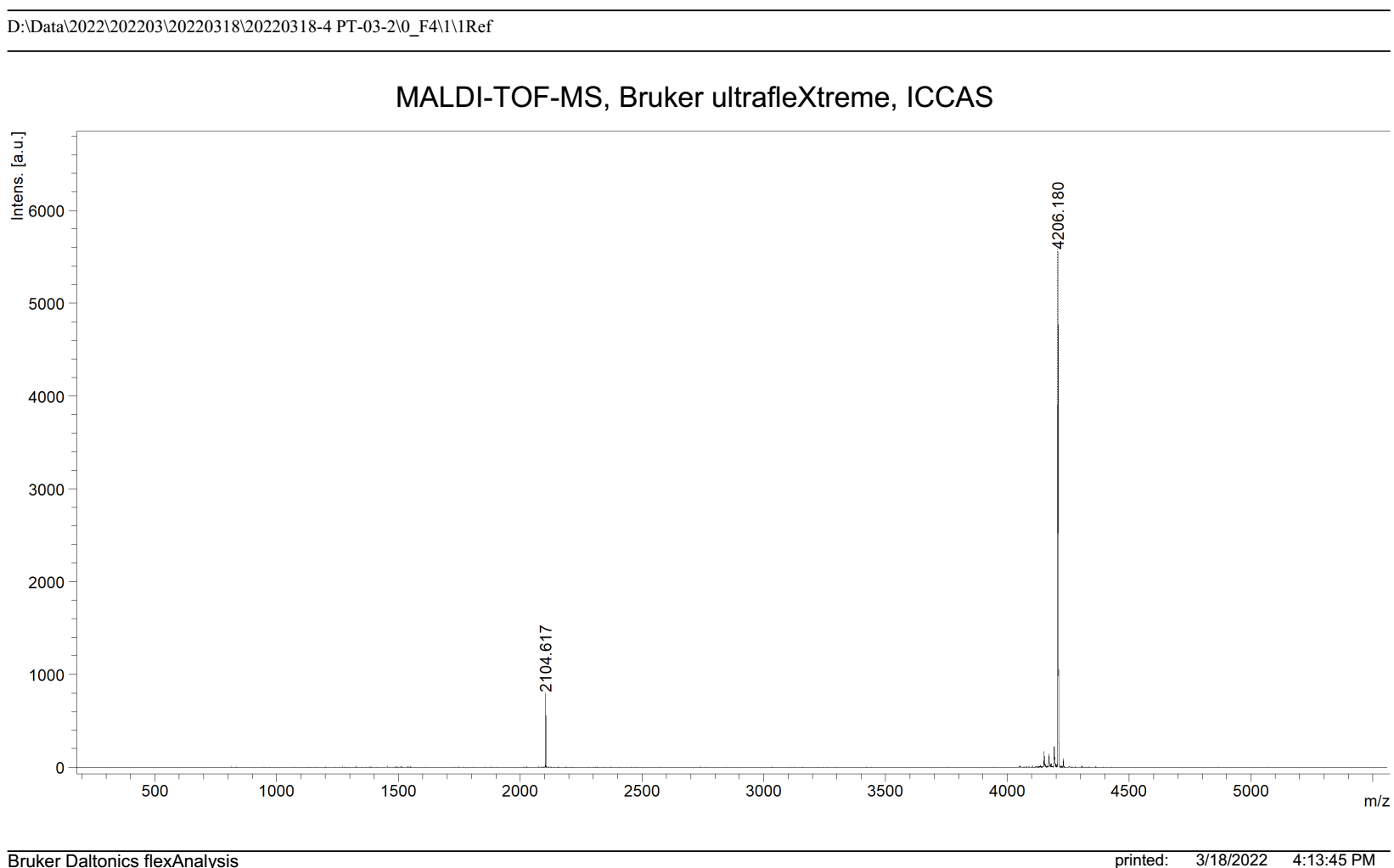


P-03: GRKKRRQRRR-HHHHHH-K(C_18_)-PIHRYHA


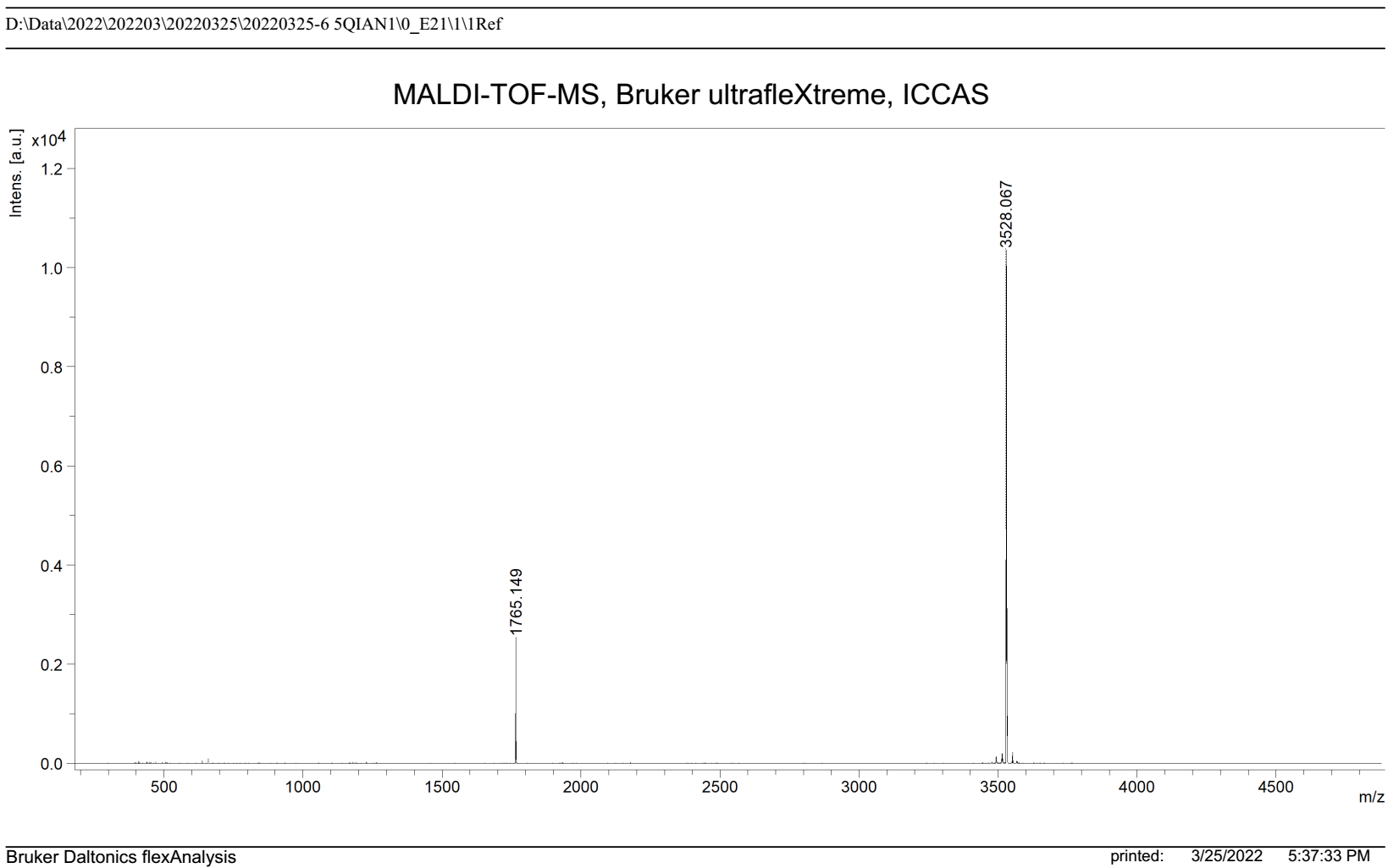


P-04: GRKKRRQRRR-LLHHLLHHLLHH-K(C_18_)-PIHRYHA


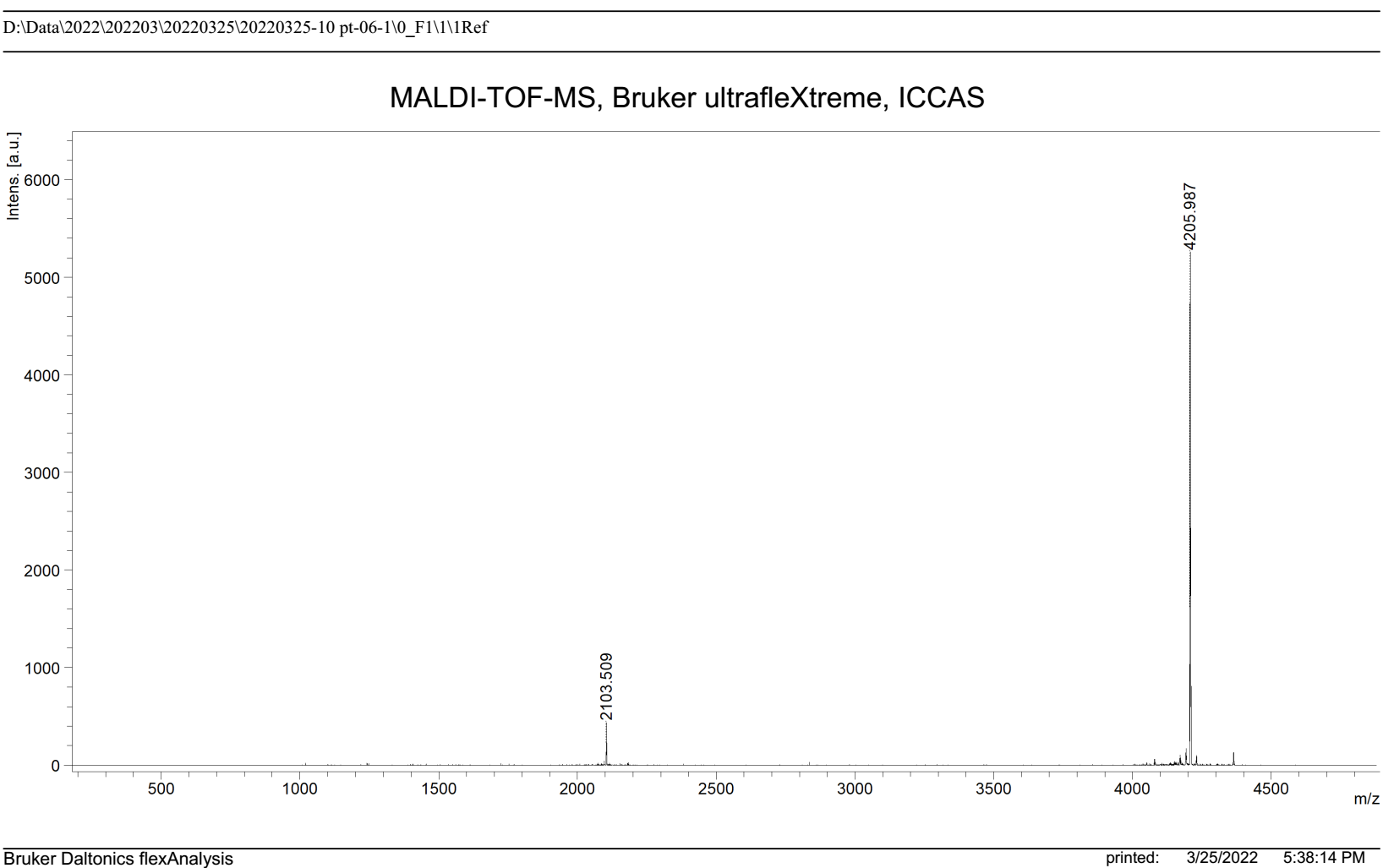


P-05: GRKKRRQRRR-LLHHLLHHLLHH-K(C_18_)


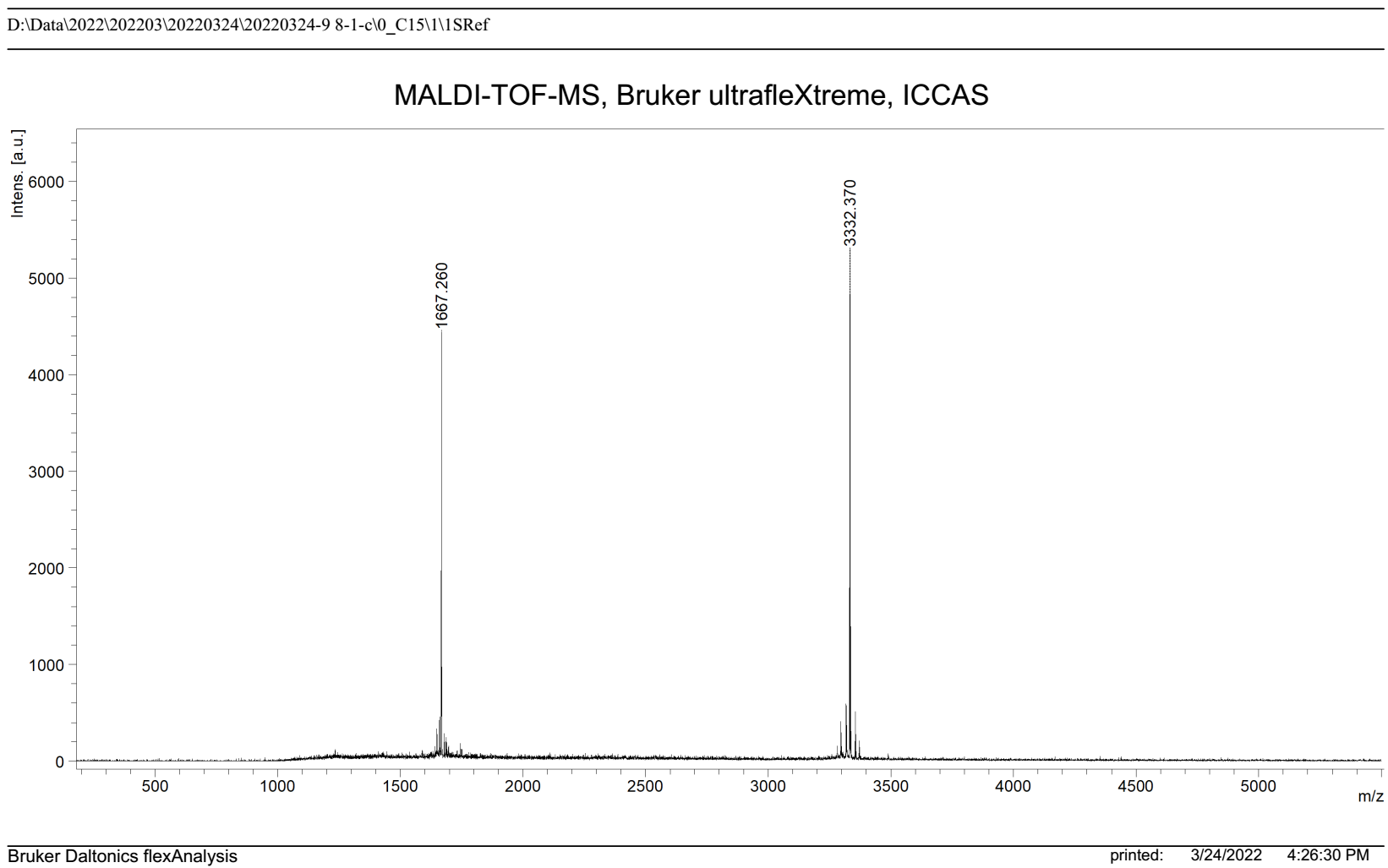


HAIYPRH


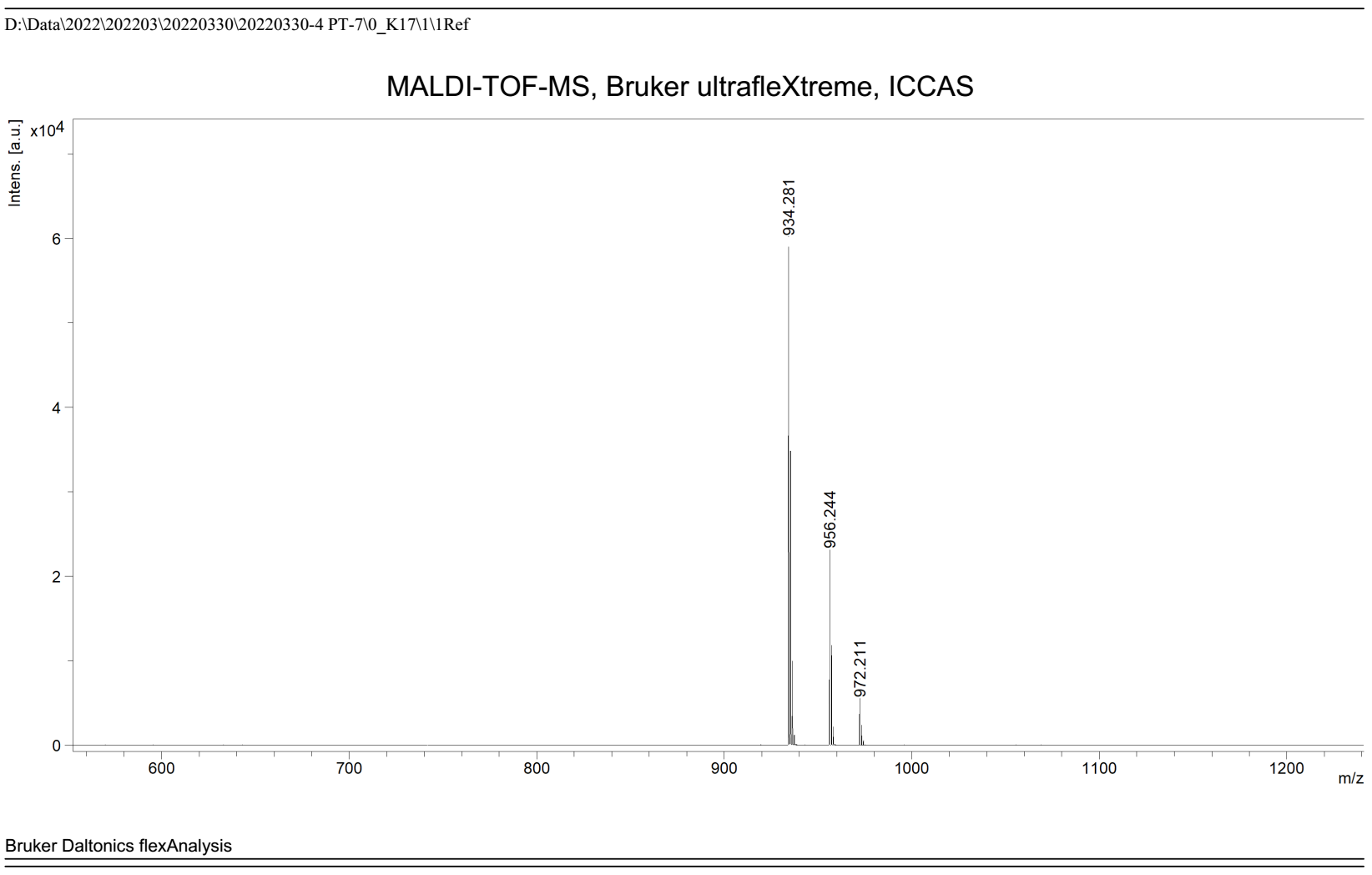


Fig. S5 HPLC of peptides in this paper.

PT-01: GRKKRRQRRR-HHHHHH-K(C_18_)-HAIYPRH





P-02: GRKKRRQRRR-LLHHLLHHLLHH-K(C_18_)-HAIYPRH





P-03: GRKKRRQRRR-HHHHHH-K(C_18_)-PIHRYHA





P-04: GRKKRRQRRR-LLHHLLHHLLHH-K(C_18_)-PIHRYHA





P-05: GRKKRRQRRR-LLHHLLHHLLHH-K(C_18_)





T7: HAIYPRH
